# Narcissistic Enough to Challenge: The Effect of Narcissism on Change-Oriented Organizational Citizenship Behavior

**DOI:** 10.3389/fpsyg.2021.792818

**Published:** 2022-02-09

**Authors:** Yi Lang, Hongyu Zhang, Jialin Liu, Xinyu Zhang

**Affiliations:** ^1^International Business School, Beijing Foreign Studies University, Beijing, China; ^2^CUFE Business School, Central University of Finance and Economics, Beijing, China; ^3^THU School of Economics and Management, Tsinghua University, Beijing, China

**Keywords:** environmental uncertainty, trait activation theory, narcissism, change-oriented organizational citizenship behavior, felt responsibility for constructive change

## Abstract

During the COVID-19 pandemic, organizations need to effectively manage changes, and employees need to proactively adapt to these changes. The present research investigated when and how individual employees’ narcissism was related to their change-oriented organizational citizenship behavior. Specifically, based on a trait activation perspective, this research proposed the hypotheses that individual employees’ narcissism and environmental uncertainty would interactively influence employees’ change-oriented organizational citizenship behavior via felt responsibility for constructive change; furthermore, the effect of narcissism on change-oriented organizational citizenship behavior via felt responsibility for constructive change would be stronger when the environmental uncertainty prompted by the COVID-19 pandemic was high rather than low. Two studies were conducted to test these hypotheses: an online survey of 180 employees in mainland China (Study 1) and a field study of 167 leader–follower dyads at two Chinese companies (Study 2). The current research reveals a bright side of narcissism, which has typically been recognized as a dark personality trait, and enriches the understanding of the antecedents of change-oriented organizational citizenship behavior. This research can also guide organizations that wish to stimulate employee proactivity.

## Introduction

The COVID-19 pandemic has brought significant and far-reaching challenges to the workplace. These changes, such as remote work, virtual teamwork, and digital transformations, have presented employees with new requirements that are not addressed in formal job descriptions or employment contracts. For example, employees need to learn and master new knowledge and skills in virtual offices (Schinoff et al., 2020), and online communication styles are different from those in traditional offices (Nyberg et al., 2021). In these new situations, employees’ proactive behaviors—such as suggesting new ideas or methods to solve non-routine issues, taking initiative to improve efficiency, and taking responsibility for extra work during periods of organizational change—will be particularly valued. These behaviors have been examined under the rubric of change-oriented organizational citizenship behavior (OCB-CH), defined as “constructive efforts by individuals to identify and implement changes with respect to work methods, policies, and procedures to improve the situation and performance” (Choi, 2007, p. 469). Although traditional organizational citizenship behaviors (OCBs) are important, they may fail to address the challenges in a dynamic environment (Li et al., 2017) which presents a great deal of uncertainty and ambiguity (Simerly and Mingfang, 2000). OCB-CH, which has been characterized as personal initiative, task revision, voice, innovative behavior, and taking charge (Scott and Bruce, 1994; Frese et al., 1997; Van Dyne and LePine, 1998), should be preferred. Employees are on the front lines and thus closest to changes in the environment. Consequently, they are often best informed regarding current practices and weaknesses (Lawler, 1992), and their initiative and voice can help organizations better cope with uncertainty (Seppälä et al., 2012). Therefore, it is theoretically and practically important to explore which types of employees are more likely to demonstrate OCB-CH.

Numerous personal characteristics are associated with OCBs, including altruism (Klotz et al., 2018), agreeableness (Ilies et al., 2009), conscientiousness (Jiao et al., 2013), and compliance (Organ and Ryan, 1995). Among these studies, however, OCBs were mainly viewed in terms of maintaining and reinforcing the *status quo* (Choi, 2007). Typical examples include cooperating with coworkers, helping coworkers accomplish their jobs, and voluntarily working beyond job requirements (Borman and Motowidlo, 1993). Compared with these behaviors, OCB-CH embodies not only the “prosocial” and “proactive” elements but also the “changing” element, and thus requires employees to welcome changes, take risks and display self-confidence. Therefore, OCB-CH may be associated with different personal characteristics.

Narcissism is an individual characteristic rooted in a grandiose and inflated self-view that desires attention and recognition (Campbell et al., 2005). It was originally regarded as a mental disorder (American Psychiatric Association, 1994) and associated with symptoms such as depression, anxiety, hostility, and paranoia (Miller et al., 2010). However, scholars later found that narcissism was a trait commonly encountered in individuals (Rosenthal and Pittinsky, 2006). Therefore, a distinction was made between grandiose and vulnerable narcissism by separating the extremely dysfunctional aspects of narcissism (Miller et al., 2011). Most narcissism research in organizational contexts has focused on grandiose rather than vulnerable narcissism (e.g., Chatterjee and Hambrick, 2007; Reina et al., 2014; Zhu and Chen, 2015). Consistent with these literature, we define narcissism in this research as grandiose narcissism (hereafter, simply “narcissism”), which is associated with grandiose self-image (Krizan and Herlache, 2018), enhanced sense of entitlement and superiority (Campbell and Campbell, 2009), desire and search for social admiration (Back et al., 2013), propensity to display dominance (Miller et al., 2011), aggressiveness and assertiveness, and determined will (Wink, 1991). To repeatedly reinforce their self-image (Kohut and Wolf, 1986), narcissists often undertake challenging tasks in a bold and risky way (Fay and Sonnentag, 2012) so that their behaviors can be visible and admired (Chatterjee and Hambrick, 2007). Because individuals displaying OCB-CH is intended to induce change, take charge, and improve situations and performance (Bettencourt, 2004), OCB-CH may be associated with narcissism. To date, however, little research has investigated the relationship between narcissism and OCB-CH.

To address this gap in the literature, we relied on trait activation theory (TAT; Tett and Guterman, 2000) and developed a theoretical model to depict when and how narcissism leads to high levels of OCB-CH. According to TAT, the influence of personality traits on behaviors is contingent upon situation trait relevance, and a personality trait is more strongly related to behavior when a situation provides cues for the expression of that trait (Lievens et al., 2006). For instance, the personality trait of proactivity will be more likely to manifest itself in a person’s behavior when the context allows for proactivity (Crant, 1995). The current research considers the environmental uncertainty engendered by the COVID-19 pandemic to be a highly relevant situation for narcissism and proposes that narcissists are more likely to feel responsible for constructive change and demonstrate OCB-CH in such situations. Environmental uncertainty is defined as “an individual’s perceived inability to predict an organization’s environment accurately” because of a “lack of information” or “an inability to discriminate between relevant and irrelevant data” (Milliken, 1987, p. 136). Due to the COVID-19 pandemic, work procedures, management systems, and work team coordination all have suffered ambiguity and uncertainty, which gives narcissistic employees an ideal opportunity to demonstrate their uniqueness by taking responsibility for reform and change. Therefore, this research proposes that narcissism and environmental uncertainty have an interactive effect on employees’ felt responsibility for constructive change (FRCC), which is a motivational state in which individuals feel a personal obligation to bring about constructive change at work (Hackman and Oldham, 1976; Morrison and Phelps, 1999). FRCC inspires proactive behaviors, and empirical evidence supports a positive relationship between FRCC and OCB-CH (e.g., Loìpez-Domiìnguez et al., 2013). Accordingly, we propose that the positive effect of narcissism on OCB-CH via FRCC will be stronger when environmental uncertainty is high rather than low.

This research makes three important contributions to the literature. First, our findings indicate that narcissism is a new antecedent of OCB-CH. Previous studies on the antecedents of OCB-CH have mostly focused on leadership styles and work contexts and found that strong vision, innovative climate, supportive leadership (Choi, 2007), transformational leadership (Loìpez-Domiìnguez et al., 2013), and empowering leadership (Li et al., 2016) exert significant influence on OCB-CH. Previous studies have also identified individual differences such as role breadth self-efficacy (Loìpez-Domiìnguez et al., 2013), personal values, sense of power (Seppälä et al., 2012), and promotion focus (Simo et al., 2016) as antecedents of OCB-CH. This research identifies a new antecedent of OCB-CH and enriches related research by revealing that bold, self-inflated personality traits can also lead to positive work behavior.

Second, we investigate the “bright side” of a commonly recognized “dark” personality trait. Previous research on narcissism has largely focused on its negative outcomes—such as counterproductive work behaviors, envy, and emotional exhaustion (for a review, see Braun, 2017)—whereas relatively little attention has been paid to its positive aspects (Smith and Webster, 2018; Mao et al., 2020). In recent decades, an increasing number of researchers have begun to investigate the latter question to gain a more comprehensive view of this personality trait (Goncalo et al., 2010; Hirschi and Jaensch, 2015; Nevicka et al., 2016; Den Hartog et al., 2020). The current research responds to this new direction and contributes to the literature by finding a new association between narcissism and desirable work outcomes.

Relatedly, we propose a critical boundary condition of environmental uncertainty in examining the effect of narcissism. Previous research found the “bright side/dark side” duality of narcissism (Hogan and Hogan, 2001; Watts et al., 2013), highlighting the need for more nuanced perspectives on its effects (Liu et al., 2017). One important area of investigation involves discovering under what circumstances narcissism exerts a stronger or weaker effect on employee behavior. Environmental uncertainty is particularly relevant to narcissism in that it provides the “opportunity for glory” (Wallace and Baumeister, 2002, p. 820), activates narcissists’ desires for self-affirmation and self-enhancement, and will elevate the behavioral effects of employee narcissism. Previous studies have suggested that narcissists are more active in ambiguous and unpredictable situations (Brunell et al., 2008) and perform better in crises (Wallace and Baumeister, 2002). When they have the opportunity for self-enhancement, narcissists are more likely to take initiative and to become highly visible by engaging in challenging or bold behaviors (Chatterjee and Hambrick, 2007). Utilizing the unique situation of the COVID-19 pandemic as a research background, we propose that the effect of narcissism on individuals’ sense of responsibility for change—and, subsequently, proactive OCB-CH—will be stronger when the environmental uncertainty prompted by the COVID-19 pandemic is high.

Third, we highlight a new mechanism in explaining the positive effect of narcissism on positive organizational behavior (i.e., OCB-CH). We find that narcissism interacts with environmental uncertainty to make employees to perceive a sense of responsibility to take initiative and lead organizational change. Few studies have investigated the underlying motivational state for the positive effects of narcissism (Mao et al., 2020). This study proposes FRCC as a new underlying mechanism.

## Theory and Hypothesis

### Employee Narcissism and Change-Oriented Organizational Citizenship Behavior: A Trait Activation Theory Lens

We build on TAT to explain why narcissism is a trait that is likely to be associated with OCB-CH in an environment characterized by uncertainty. We also explain the mechanisms of this relationship. TAT can be broadly applied to a range of personality traits, including narcissism (Liu et al., 2017). It is an interactionist theory that posits that although personality traits are relatively stable and guide behaviors in general, they do not manifest equally across all situations (Liu et al., 2017) and certain situations may strengthen or weaken the impacts of personality traits on behavior (Tett and Burnett, 2003). Although “we see traits by what we see people do,” we only see strong personality–behavior connections for traits that are activated and manifested (Tett and Burnett, 2003, p. 502). Notably, situation relevance—that is, “the qualitative feature of situational demands that increase the likelihood that individuals will demonstrate more of a particular behavior over other behaviors” (Oliver et al., 2016, p. 1997)—serves as a moderator that enables the expression of trait-relevant behavior. When situations present cues for expressing trait-relevant behaviors, traits are activated, and the personality–behavior connection becomes strong (Tett and Guterman, 2000).

Narcissism is a personality trait that is characterized by “an inflated sense of self and is preoccupied with having that self-view continually reinforced” (Chatterjee and Hambrick, 2007, p. 353). Although narcissism is generally considered as a negative or even psychopathological trait (for a review, see Braun, 2017), it has also been observed that many good leaders are, in practice, narcissistic. For instance, Brunell et al. (2008) found that narcissists emerged as group leaders in leaderless group discussions. Moreover, as Morf et al. (2011) noted, narcissists believe that “if opportunity [exists] for promotion or demonstration of the grandiose and superior self, then self-affirm, self-promote and self-enhance!” (p. 402), highlighting the importance of context and opportunity in igniting the manifestation of narcissism. Prior studies have also demonstrated that narcissism has mixed impacts on behaviors (Liu et al., 2017). In particular, the relationship between narcissism and OCB-CH may be positive, given the proactive aspect of narcissism (Hirschi and Jaensch, 2015), or negative/insignificant, given the egocentric aspect of narcissism (Peterson et al., 2012).

Trait activation theory is a suitable umbrella theory to guide our conceptual framework because it introduces situations as important boundary conditions in understanding the relationship between personality traits and behaviors. According to this theoretical lens, in order to build a positive relationship between narcissism and OCB-CH, we should identify situational cues that are relevant to the proactive component of narcissism. Trait-relevant cues may exist at the task, social, and organizational levels (Tett and Burnett, 2003). In this research, we choose one organizational-level cue: environmental uncertainty. Below, we explain how narcissism and environmental uncertainty jointly influence OCB-CH via FRCC.

### The Interaction of Employee Narcissism and Environmental Uncertainty on Felt Responsibility for Constructive Change

Felt responsibility for constructive change refers to “the extent to which an individual feels personally responsible for continually redefining performance (i.e., doing things better), rather than solely performing his or her own task well according to current performance standards (i.e., doing the job right)” (Fuller et al., 2006, p. 1092). It reflects a willingness to make an exceptional effort within one’s organization and makes individuals more likely to engage in extra-role behaviors (Ran and Zhou, 2020). Several elements of narcissism should be positively linked to FRCC, and we refer to those elements as the proactive side of narcissism.

First, narcissistic employees have inflated self-views and are extremely self-confident (Campbell et al., 2002; Martin et al., 2016). They believe that they are more knowledgeable and experienced than others and that they should be dominant in leading organizational change (Zhu and Chen, 2015). Relatedly, narcissistic employees have a strong need for power and control, prefer to take a dominant role at work, and long for others’ compliance or even worship. They are thus likely to take charge and be the “first mover” or the “savior” of their organization, and shoulder the responsibility for organizational change (Spurk and Hirschi, 2018).

Second, narcissistic employees need continuous reaffirmation of their superiority (Chatterjee and Hambrick, 2007). Merely fulfilling their job requirements will not satisfy their desire to gain admiration (Wallace and Baumeister, 2002). They enjoy gaining attention and recognition by standing out from the ordinary people and achieving distinction. Initiating change is a visible way to demonstrate superiority. Narcissistic employees are thus willing to explore opportunities to advance the *status quo* (Campbell et al., 2011; Ha et al., 2020). Prior research has found that narcissistic leaders present their followers with a vision of a future that is far superior to the *status quo* (Ha et al., 2020). In a similar vein, we propose that narcissistic employees will be more motivated to take FRCC.

Finally, narcissistic employees are more willing to take risks (Campbell et al., 2004). Change and reform always involve risk (Brouthers et al., 2002). Risk-averse employees may find it difficult to overcome their fear of failure and therefore may be extremely reluctant to initiate change (Heavey et al., 2010). However, for narcissistic employees, the promise of public praise encourages them to take risky actions (Campbell et al., 2004). Narcissistic CEOs are considered to be extraordinarily useful, and even necessary in pioneering organizational and industrial change (Maccoby, 2000).

In sum, based on an inflated self-view and driven by the needs for power and self-affirmation, narcissistic employees are likely to feel more responsible for constructive change, demonstrate greater confidence in the face of uncertainty, and experience less fear regarding potential risks. However, according to TAT, the manifestation of narcissism may vary across situations. We propose that environmental uncertainty is a crucial environmental cue in determining the strength of the relationship between narcissism and FRCC. As suggested by Wallace and Baumeister (2002), “when there is an opportunity for glory, narcissists will shine, but they will underperform when the opportunity for glory is not available” (p. 1664). Environmental uncertainty provides narcissists with an excellent opportunity for self-enhancement. Narcissists’ motivation to demonstrate superiority and gain attention “can additionally be prompted by situational cues” (Back et al., 2013, p. 1016). In particular, when environmental uncertainty is high, narcissistic employees will be more likely to feel responsible for constructive change. This is because environmental uncertainty is relevant to narcissism, especially to elements such as inflated self-view, need for power, self-affirmation, self-confidence, and willingness to take risks.

Environmental uncertainty includes uncertainties at three levels: (1) state uncertainty, or uncertainty about how the environment will change; (2) effect uncertainty, or uncertainty about how environmental changes will impact the organization; and (3) response uncertainty, or uncertainty about the consequences of organizational responses to environmental change (Ellis and Shpielberg, 2003). These uncertainties are relevant to the above-mentioned elements of narcissism and are thus expected to strengthen the relationship between employee narcissism and FRCC.

At the level of state uncertainty, environmental uncertainty is relevant to narcissism in that it provides employees opportunities to demonstrate superiority for self-affirmation. In a stable and predictable environment, attention is not easily attracted through daily routines; there is little room for employees to show their superiority (Chatterjee and Pollock, 2016) and therefore no glory to be gained (Wallace and Baumeister, 2002). However, in an environment that is full of uncertainty, employees’ behaviors are more likely to be observed, and they have opportunities to shine (Brunell et al., 2008). Employees are thus motivated to exhibit narcissism (Wisse et al., 2015), gaining self-affirmation by “undertaking challenging or bold tasks that are highly visible to a respected audience” (Chatterjee and Hambrick, 2007, p. 354). Prior research has found that narcissists perform better in crises than in stable environments (Wallace and Baumeister, 2002). In a similar vein, we argue that the relationship between narcissism and FRCC should be stronger when environmental uncertainty is high.

At the level of effect uncertainty, environmental uncertainty is relevant to narcissism because it requires a willing hero who dares to guide others. In a stable and predictable environment, everyone has a clear understanding of their roles and responsibilities, and there are few situations characterized by ambiguity or lack of direction. Thus, no such heroes are needed (Venus et al., 2019). However, in an environment full of uncertainty, narcissism is valued because people who believe that they should dominate (Zhu and Chen, 2015), be leaders (Judge et al., 2006), firmly pursue goals despite adversity (Rosenthal and Pittinsky, 2006), and behave assertively rather than cautiously and indecisively (Leckelt et al., 2015) can help organizations (Wallace and Baumeister, 2002). Uncertainty has been found to enhance the preference for narcissistic leaders, as the overconfidence and dominance of narcissistic leaders satisfy the demand for “strength and toughness” in uncertain contexts (Nevicka et al., 2013, p. 371). Therefore, narcissism will be activated and demonstrated, and the relationship between narcissism and FRCC should, accordingly, be stronger.

At the level of response uncertainty, environmental uncertainty is relevant to narcissism because it highlights the importance of risk-taking and self-confidence. In a stable and predictable environment where rules are definite, organizations and their members are certain of the consequences of their behaviors and rarely take risks. Therefore, people are encouraged to behave in a safe way, rather than in the aggressive manner that is characteristic of narcissists (Heavey et al., 2010). However, in an environment that is full of uncertainty, the consequences of organizational responses to the environment are ambiguous, and decisions in the organization are often risky. Hence, employees need to take risks to do their jobs (Brouthers et al., 2002). Narcissism enables employees to disrupt the *status quo*, make ambitious plans, and believe that their decisions will lead to the best outcomes (Zhu and Chen, 2015). Therefore, when environmental uncertainty is high, narcissism becomes necessary and encouraged, and the relationship between narcissism and FRCC should in turn become stronger.

In sum, environmental uncertainty provides relevant cues to narcissism. Narcissism, which is characterized by inflated self-views, the need for power, self-affirmation, self-confidence, and risk-taking, can be activated by environmental uncertainty, strengthening its relationship with FRCC. Thus, we propose:

Hypothesis 1: Employee narcissism and environmental uncertainty will interactively influence employee’s FRCC, in such a way that employee narcissism will be more positively related to his/her FRCC when environmental uncertainty is high.

### The Interaction of Employee Narcissism and Environmental Uncertainty on Change-Oriented Organizational Citizenship Behavior via Felt Responsibility for Constructive Change

Change-oriented organizational citizenship behavior is a form of proactive behavior, defined as constructive efforts by individuals to identify and implement changes to work methods, policies, and procedures to improve situations and performance (Choi, 2007). The most proximal and direct predictors of proactive behavior are motivational processes (Bindl and Parker, 2011). FRCC has been suggested as the motivation, or “reason to,” that explains valence, or “why” individuals engage in proactive behaviors such as OCB-CH (Fuller et al., 2012, p. 1054). It reflects individuals’ internalized goals that are deemed to be of great value (Deci and Ryan, 2002) and thus can greatly determine behaviors.

First, FRCC reflects individuals’ internal intentions to redefine and reform performance (Fuller et al., 2006), as opposed to being assigned responsibility. It is thus an identified form of self-regulation and is associated with a great sense of personal accomplishment and satisfaction achieved through initiating change (Morrison and Phelps, 1999). For individuals who possess a strong sense of FRCC, it is of positive valence for them to engage in OCB-CH. In contrast to affiliative types of OCB, OCB-CH includes challenging and risk-taking behaviors, such as personal initiative, task revision, voice, and taking charge. Individuals’ sense of obligation concerning change leads them to question current practices and challenge the *status quo*, rather than simply behaving in a conscientious and compliant manner.

Second, previous studies have suggested that FRCC motivates individuals to more thoroughly process work-related information, thus helping them identify possible areas for improvement or reform (Fuller et al., 2006). This can make it more likely for individuals to initiate OCB-CH. In addition, OCB-CH involves the ability to take charge and assume the risks of not being welcomed and, ultimately, of failure. Individuals with high levels of FRCC have a sense of ownership over their work and possess the confidence to take on a dominant role in challenging the *status quo*. This increases their willingness to take risks in order to accomplish new achievements in their tasks, making them more likely to exhibit OCB-CH.

Moreover, empirical evidence has been found for positive relationships between FRCC and OCB-CH (e.g., Loìpez-Domiìnguez et al., 2013), taking charge (Morrison and Phelps, 1999; Parker and Collins, 2010), voice (Chamberlin et al., 2017), continuous improvement (Fuller et al., 2006), and innovation (Parker and Collins, 2010). Therefore, we propose:

Hypothesis 2: Employee FRCC is positively related to his/her change-oriented OCB.

Combining Hypotheses 1 and 2, this study further proposes that employee narcissism and environmental uncertainty will have an interactive effect on OCB-CH via FRCC. Narcissists’ inflated self-view, strong need for competence and dominance, and pursuit of praise and status via risk taking and exploration generate feelings of accountability that lead them to initiate change and reform. We expected that this relationship would be moderated by environmental uncertainty, because the variable and unpredictable character of the environment is relevant to narcissists’ need for self-affirmation and self-enhancement. Uncertain situations are likely to activate narcissists’ desires to be highly visible, and to generate a perceived obligation to initiate change. Subsequently, FRCC should lead to their constructive efforts to identify and implement changes to work methods, policies, and procedures aimed at improving their organization. Therefore, we propose:

Hypothesis 3: Employee narcissism and environmental uncertainty interactively influence change-oriented OCB via FRCC.

**FIGURE 1:**
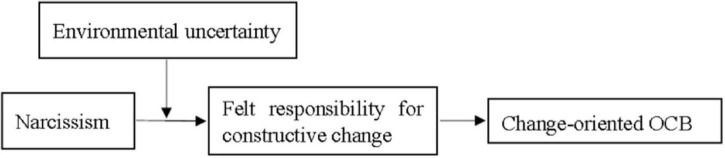
Theoretical model.

The theoretical model of the current study is shown in Figure 1.

## Materials and Methods

We conducted two studies to test our hypotheses. Study 1 was an online survey we administered when COVID-19 broke out in Hubei province. Because at that time, COVID-19 was mostly found in Hubei province and had not spread to other provinces, we expected that employees working in Hubei would face higher levels of uncertainty compared to employees working elsewhere. Taking this opportunity, we adopted an objective indicator to capture environmental uncertainty: work locations were coded as “1” for Hubei province (i.e., high environmental uncertainty) and as “0” for other provinces (i.e., low environmental uncertainty). Study 2 used a leader-follower matched data to replicate the results of Study 1 in two high-tech companies in Beijing, China. To complement Study 1, Study 2 used a validated scale to measure environmental uncertainty in a more refined way and collected data from different sources to avoid potential common method variance (CMV).

### Study 1

#### Sample and Procedure

We recruited 185 employees in China via Credamo (a professional survey platform recognized by top international journals; Jin et al., 2021). The survey was conducted in January 2020, 1 month after the outbreak of the COVID-19 epidemic, which was particularly serious in Hubei province. Credamo randomly distributed questionnaires in China (excluding Hong Kong, Macao, and Taiwan), and targeted employees who had more than 6 months’ work experience (Harris et al., 2014). The questionnaire passed Credamo’s audit, which guaranteed that it would not cause negative psychological effects on participants. Also, at the beginning of the questionnaire, we briefly informed participants that the survey was about organizational management and employee working conditions during the epidemic, their participation was voluntary, and their responses would only be used for academic purposes. Finally, 180 valid questionnaires were obtained.

The proportion of male and female participants was the same (50%). The average age of the respondents was 29.63 (*SD* = 5.99), and the average organizational tenure was 7.37 years (*SD* = 5.82). The respondents had relatively high levels of education (72.78% had a bachelor’s degree or higher). Most of the respondents were general staff (40.56%) or lower-level managers (37.78%) and from private enterprises (46.11%).

#### Measures

We applied mature scales to measure narcissism, FRCC, and OCB-CH. We translated these scales from English to Chinese following the translation and back-translation procedure (Brislin, 1980). Two bilingual research assistants who were blind to the nature of the study and hypotheses completed the translations. Disagreements were resolved through consensus-based discussion among the authors, translators, and other bilingual researchers. A seven-point scale was used for all the questionnaires, ranging from “1” (strongly disagree) to “7” (strongly agree).

##### Narcissism

We used the 16-item Narcissistic Personality Inventory (NPI-16; Ames et al., 2006) to measure narcissism. NPI-16 was proved to be one of the two scales that have the strongest match with expert ratings of grandiose narcissism (Miller et al., 2014) and thus is commonly used to measure grandiose narcissism (Nevicka et al., 2011a,b). The NPI-16 assesses the tendency to hold grandiose self-views along with corresponding behavioral propensities and is meant to assess subclinical aspects of narcissism. A sample item was “I like to be the center of attention.” Cronbach’s Alpha was 0.88 in this study.

##### Felt Responsibility for Constructive Change

Following Choi (2007), we measured FRCC by two items developed by Morrison and Phelps (1999): “I feel a personal sense of responsibility to bring about change at work” and “it’s up to me to bring about improvement in my workplace.” Cronbach’s Alpha was 0.79 in this study.

##### Change-Oriented Organizational Citizenship Behavior

We adopted the 4-item scale from Choi (2007) to measure OCB-CH. A sample item was, “I often suggest changes to unproductive rules or policies.” Cronbach’s Alpha was 0.88 in this study.

##### Environmental Uncertainty Brought on by the COVID-19 Pandemic

As the pandemic broke out in Hubei province in January 2020, most cases of COVID-19 in China were in Hubei, and the government imposed various measures to facilitate the fight against its transmission in this province. The lockdown of cities and workplaces, transition to online work, use of remote workspaces, prolonged suspensions of work, and undecided work-resumption timing brought about great environmental uncertainty. Employees in Hubei thus faced higher levels of environmental uncertainty compared with employees in other provinces. For these reasons, the current survey used whether an employee’s workplace was in Hubei to indicate the level of environmental uncertainty brought on by the COVID-19 pandemic: “1” (Hubei) represents a high level of environmental uncertainty and “0” (other provinces) represents a low level of environmental uncertainty.

##### Control Variables

Past literature indicates that employees’ gender, age, education level, and organizational tenure may influence their levels of narcissism, tendencies to take initiatives, and tendencies to conduct OCB-CH to a certain extent. We also controlled for organization type and job level because employees in different types of organizations and job levels may face different levels of environmental uncertainty and feel different levels of responsibility for change. The type of industry was also controlled because different industries have experienced varying levels of environmental changes during the pandemic, and employees may feel different levels of responsibility for change and for conducting OCB-CH.

#### Analytical Approach

We first performed confirmatory factor analysis (CFA) using AMOS 24.0, and then applied a CMV test. After these primary analyses, we used SPSS 26.0 to conduct regression analysis to test Hypothesis 1 and Hypothesis 2, and tested the overall model (Hypothesis 3) via Monte Carlo (MC) simulation using Mplus 7.4.

#### Confirmatory Factor Analysis

To test factorial validity and the construct distinctiveness of narcissism, FRCC, and OCB-CH, we conducted CFA. Owing to the limited sample size, this study used the factorial algorithm method of item parceling (Rogers and Schmitt, 2004) before conducting CFA. Two item parcels were created for narcissism. These item parcels were considered indicators of the construct. In addition, all items of FRCC and OCB-CH were viewed as indicators of the two constructs. As demonstrated in Table 1, the hypothesized three-factor model provided a good fit, with all the fit indices within acceptable levels (χ2/df = 2.17, RMSEA = 0.08, CFI = 0.97, TLI = 0.95, IFI = 0.97). After examining the fit of all the alternative models, the three-factor model offered a superior fit for the data.

**TABLE 1:** Study 1 results of confirmatory factor analysis.

Model	Factors	χ2	df	△χ2	RMSEA	CFI	TLI	IFI
Baseline	Three factors: N, FRCC, OCB-CH	36.83	17		0.08	0.97	0.95	0.97
Alternatives								
Model 1	Two factors: N + FRCC, OCB-CH	125.29	19	88.46**	0.18	0.84	0.77	0.85
Model 2	Two factors: N + OCB-CH, FRCC	99.07	19	62.24**	0.15	0.88	0.83	0.88
Model 3	Two factors: N, FRCC + OCB-CH	82.37	19	45.54**	0.14	0.91	0.86	0.91
Model 4	One factor: all variables combined	145.71	20	108.88**	0.19	0.81	0.74	0.82

**p < 0.01. N, narcissism; FRCC, felt responsibility for constructive change; OCB-CH, change-oriented OCB.

#### Common Method Variance Test

Because we adopted the questionnaire survey method, and all the variables were answered by a single person, there may be a CMV problem. We applied the Harman single-factor test to determine the level of CMV in the study. The results showed that the variance of the first common factor accounted for was 31.97%, far below the 50% standard (Yong and Pearce, 2013), indicating that there is no serious CMV problem among the measured variables.

#### Descriptive Analysis Results

Table 2 presents the means, standard deviations, zero-order correlations, and internal consistency alphas for all the variables. Consistent with our hypotheses, narcissism was positively and significantly related to FRCC (*r* = 0.25, *p* < 0.01), and FRCC was positively and significantly related to OCB-CH (*r* = 0.60, *p* < 0.01).

**TABLE 2:** Study 1 descriptive statistics and correlation matrix.

	*M*	*SD*	1	2	3	4	5	6	7	8	9	10	11	12
(1) Gender (0 = male, 1 = female)	0.50	0.50												
(2) Age	29.63	5.99	–0.03											
(3) Education level	3.69	0.86	0.18*	0.18										
(4) Organization tenure	7.37	5.82	0.001	0.80**	−0.18*									
(5) Organization type (0 = non-state owned, 1 = state-owned)	0.27	0.44	0.05	–0.07	0.22**	–0.12								
(6) Job level	1.83	0.82	0.000	0.18*	0.26**	0.30**	–0.03							
(7) Industry 1	0.18	0.38	0.09	0.14^†^	−0.21**	0.15*	0.02	–0.10						
(8) Industry 2	0.35	0.48	–0.01	−0.15*	0.05	−0.20**	0.11	0.05	−0.34**					
(9) Narcissism	4.34	0.82	–0.02	0.02	0.09	0.02	0.16*	0.29**	0.01	–0.04	(0.88)			
(10) Environmental uncertainty	0.35	0.48	0.12^†^	0.06	−0.25**	0.11	−0.13^†^	−0.14^†^	0.02	–0.10	−0.17*			
(11) Felt responsibility for constructive change	5.67	0.95	−0.14^†^	0.12^†^	0.12	0.17*	0.12	0.17*	–0.06	0.09	0.25**	–0.07	(0.79)	
(12) Change-oriented OCB	5.42	1.02	−0.15*	0.10	0.10	0.12	0.13^†^	0.31**	–0.05	0.16*	0.43**	−0.14^†^	0.60**	(0.88)

*Coefficient alphas are reported in parentheses along the diagonal.*

^†^p < 0.1; *p < 0.05; **p < 0.01.

*Employee education level: 1, junior high school or below; 2, high school or technical school; 3, junior college; 4, bachelor’s degree; 5, master’s degree; 6, PHD.*

*Job level: 1, general staff; 2, lower-level manager; 3, middle-level manager; 4, top-level manager.*

*Industries are classified into manufacture industry, service industry, and other industries. Two dichotomous variables (industry 1, industry 2) were created to differentiate the three industries.*

#### Hypothesis Testing

**FIGURE 2:**
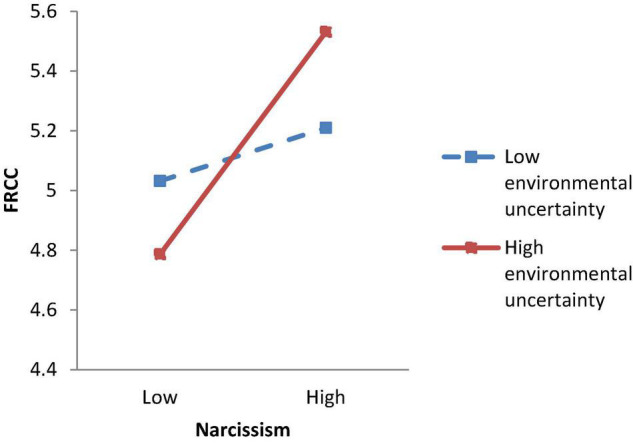
Study 1 moderating effect of environmental uncertainty on the relationship between narcissism and felt responsibility for constructive change.

We applied regression analysis to test the hypotheses. Hypothesis 1 proposed that narcissism and environmental uncertainty would have an interactive effect on FRCC. As Model 3 of Table 3 shows, the interaction term between narcissism and environmental uncertainty was significantly related to FRCC (γ = 0.36, *p* < 0.05). Figure 2 and simple slope tests show that the relationship between narcissism and FRCC was significant when environmental uncertainty was high (simple slope = 0.50, *p* < 0.01), but insignificant when environmental uncertainty was low (simple slope = 0.10, n.s.). Thus, Hypothesis 1 was supported. Hypothesis 2 proposed that FRCC would be positively related to OCB-CH. As demonstrated in Model 5 of Table 3, FRCC was significantly related to OCB-CH (γ = 0.59, *p* < 0.01). Therefore, Hypothesis 2 was supported.

**TABLE 3:** Study 1 regression results.

	Felt responsibility for constructive change						Change-oriented OCB			
	Model 1		Model 2		Model 3		Model 4		Model 5	
	b	SE	b	SE	b	SE	b	SE	b	SE
***Control variables***										
Gender	−0.28*	0.14	−0.27*	0.13	−0.25^†^	0.14	−0.32*	0.14	–0.16	0.12
Age	–0.01	0.02	–0.01	0.02	–0.004	0.02	0.01	0.02	0.01	0.02
Education	0.12	0.09	0.13	0.09	0.13	0.09	0.01	0.09	–0.06	0.08
Organization tenure	0.04^†^	0.02	0.04*	0.02	0.04	0.02	0.01	0.02	–0.01	0.02
Organization type	0.25	0.16	0.17	0.16	0.16	0.16	0.32^†^	0.17	0.17	0.14
Job level	0.08	0.10	–0.01	0.10	–0.03	0.10	0.36**	0.10	0.31**	0.08
Industry 1	–0.04	0.20	–0.05	0.19	–0.04	0.19	0.10	0.21	0.13	0.17
Industry 2	0.21	0.16	0.25	0.15	0.24	0.15	0.32*	0.16	0.20	0.14
***Independent variables***										
Narcissism			0.25**	0.09	0.28**	0.09				
Environmental uncertainty					0.04	0.15				
***Mediator***										
Felt responsibility for constructive change									0.59**	0.07
***Interaction***										
Narcissism × environmental uncertainty					0.36*	0.18				
*R* ^2^	0.11		0.15		0.17		0.16		0.43	
Adj. *R*^2^	0.07		0.11		0.12		0.12		0.40	
Δ*R*^2^			0.04**		0.02^†^				0.27**	
*F*	2.57*		3.33**		3.11**		4.12**		14.11**	

^†^p < 0.1; *p < 0.05; **p < 0.01.

Hypothesis 3 proposed that narcissism and environmental uncertainty would have an interactive effect on OCB-CH via FRCC. We applied a path analysis model using Mplus 7.4 (Muthén and Muthén, 2012). The confidence interval was calculated via Monte Carlo (MC) simulation with 20,000 replications using R^1^ (Bauer et al., 2006; Preacher and Selig, 2010). Estimation of the conditional indirect effects revealed that the indirect effect of narcissism on OCB-CH via FRCC was significant when environmental uncertainty was high (*effect* = 0.17, 95%CI [0.03, 0.32]), but insignificant when environmental uncertainty was low (*effect* = 0.01, 95%CI [–0.12, 0.13]). Thus, Hypothesis 3 was supported.

#### Study 1 Discussion

In Study 1, we found that employee narcissism and environmental uncertainty had an interactive effect on OCB-CH via FRCC, such that the indirect effect was stronger when environmental uncertainty was high rather than low. However, Study 1 has several limitations. First, environmental uncertainty was measured generally via proximal variables of the respondents - geographical locations (i.e., whether or not they worked in Hubei Province) and is thus not a direct measurement. Second, both independent and dependent variables were provided by employees; thus, the results of Study 1 may suffer from common source bias. Consequently, we conducted Study 2 to reexamine our hypotheses in specific corporate settings and invited both employees and their supervisors to respond to a survey to reduce common source bias. In addition, Study 2 applied employees’ perception of technology uncertainty brought on by COVID 19 as a measurement of environmental uncertainty, because the primary uncertainty faced by high-tech companies comes from technology. Therefore, we believed that Study 2 would provide additional reliable empirical evidence for the hypotheses.

### Study 2

#### Sample and Procedure

We collected data from two high-tech companies in Beijing, China. We obtained team supervisors’ contact information through the companies’ human resources departments. We first contacted all team supervisors to explain the purpose and confidential nature of the study and invited them to voluntarily participate in the survey. We used WeChat to send links to online surveys to the supervisors and asked them to invite one of their subordinates to participate in the study. After finishing questionnaires for leaders, the supervisors sent links of the questionnaires for followers to their subordinates. To protect the confidentiality of participants, they were assigned random identification numbers so that supervisors’ and subordinates’ responses could be matched. Supervisors provided their demographic information and assessments of subordinates’ OCB-CH; subordinates provided information on their demographics, narcissism, perceived environmental uncertainty brought on by COVID-19, and FRCC.

One hundred sixty-seven supervisors and their subordinates were invited to participate in the online survey. The supervisors’ and subordinates’ responses were then matched. The final sample included only dyadic for which both the supervisors and their subordinates responded, and each of whom had more than 6 months’ organizational tenure (Harris et al., 2014). The final sample consisted of 167 leaders and their 167 corresponding subordinates. Among the supervisors, 60.47% were male and 68.27% had a bachelor’s degree or higher. The average age was 34.77 years (*SD* = 7.51) and the average organizational tenure was 9.25 years (*SD* = 5.68). Among the subordinates, 61.07% were male and 67.66% had a bachelor’s degree or higher degrees. The average age was 31.77 years (*SD* = 6.52) and the average organizational tenure was 10.39 years (*SD* = 6.61).

#### Measures

Following Study 1, we applied the same measures for narcissism, FRCC, and change-oriented OCB in Study 2 to measure these variables. In contrast to Study 1, in which we asked employees to fill out all the questionnaires, in Study 2 we invited employees to assess their own narcissism and FRCC and asked their supervisors to assess employees’ OCB-CH. The measure for environmental uncertainty was also a mature English scale, and we followed the translation and back-translation procedure (Brislin, 1980) to translate it from English to Chinese.

##### Narcissism

Consistent with Study 1, this study applied NPI-16 (Ames et al., 2006) to measure narcissism. Cronbach’s Alpha was 0.92.

##### Felt Responsibility for Constructive Change

We followed Choi (2007) and measured FRCC by two items developed by Morrison and Phelps (1999). Cronbach’s Alpha was 0.63 in the current study. As Cortina (1993) noted, “Alpha is very much a function of the number of items in a scale, it must be interpreted with the number of items in mind” (p. 102). Although the scales of the FRCC displayed alphas lower than 0.70, they were included in the analysis for several reasons. First, the number of items for this variable was only two. Second, factor analysis using principal components established the unidimensionality of the factor. Finally, the average item intercorrelation for the factor was 0.48. Accordingly, given the number of items, factor analysis, and item intercorrelations, as well as the fact that the scale was developed by the researchers, the scale was retained in the study (Cortina, 1993).

##### Change-Oriented Organizational Citizenship Behavior

We adopted the four-item scale from Choi (2007) to measure OCB-CH. A sample item was “This employee often changes the way he/she works to improve efficiency.” Cronbach’s Alpha was 0.86 in current study.

##### Environmental Uncertainty Brought on by the COVID-19 Pandemic

We applied employees’ perception of technology uncertainty brought on by COVID-19 as the measurement of environmental uncertainty. Study 2 was conducted in two high-tech companies in mainland China. Before conducting the survey, we interviewed some of the leaders and employees in these companies and found that the primary uncertainty they felt from COVID-19 was from technology uncertainty. In their views, COVID-19 would greatly shape the future of science, technology, and innovation. Uncertainty exists in research and development (R&D) of new products and services, the adoption of digital tools and techniques, and changes in work habits. We thus measured technology uncertainty as an indicator of environmental uncertainty. We created a scale measuring perceived technology uncertainty brought on by COVID-19, adapted from that of Ragatz et al. (2002). Whereas the scale of Ragatz et al. (2002) measured respondents’ general perception of technology uncertainty in a work context, we examined subordinates’ perceptions about technology uncertainty under the specific background of COVID-19 pandemic. We stated in the questionnaire, “Due to the outbreak of the COVID-19 pandemic, corporations and industries have introduced or will introduce new technology to cope with challenges brought by the disease to the workplace.” We then asked participants to rate their level of perceived technology uncertainty in terms of three aspects: the newness of the technology, the level of complexity of the technology, and the rapid/unstable change rate of the technology. Cronbach’s Alpha was 0.72 in this study.

##### Control Variables

We controlled subordinates’ demographic information: (1) gender, because the previous study suggested that men tended to be more narcissistic than women (Grijalva et al., 2015), and were more likely to challenge the *status quo* and initiate change; (2) age and organizational tenure, as these factors may moderate the influence of context and dispositional variables on OCB (Wagner and Rush, 2000); (3) education level, as it may influence individuals’ perception of the environment and tendency to make changes; (4) company, although the two firms were both high-tech companies, they may have different corporate cultures or policies that could influence employees’ felt responsibility for change and proactive behaviors.

#### Analytical Approach

As in Study 1, we performed CFA using AMOS 24.0, applied a CMV test, and then used SPSS 26.0 to conduct regression analysis to test hypotheses. Finally, the overall model was tested via Monte Carlo (MC) simulation using Mplus 7.4.

#### Confirmatory Factor Analysis

To test factorial validity and the construct distinctiveness of narcissism, FRCC, environmental uncertainty, and OCB-CH, we conducted CFA using AMOS 24.0. As in Study 1, this study used the factorial algorithm method of item parceling (Rogers and Schmitt, 2004) before conducting CFA. We created two-item parcels for narcissism. These item parcels were considered as indicators of the construct. In addition, all items of other variables were viewed as indicators of the constructs. As demonstrated in Table 4, the hypothesized four-factor model provided a good fit, with all the fit indices within acceptable levels (χ2/df = 2.27, RMSEA = 0.08, CFI = 0.93, TLI = 0.90, IFI = 0.93). After examining the fit of all the alternative models, the four-factor model offered a superior fit for the data.

**TABLE 4:** Study 2 results of confirmatory factor analysis.

Model	Factors	χ2	df	△χ2	RMSEA	CFI	TLI	IFI
Baseline	Four factors: N, FRCC, EU, OCB-CH	86.43	38		0.08	0.93	0.90	0.93
**Alternatives**								
Model 1	Three factors: N + FRCC, EU, OCB-CH	106.29	41	19.86**	0.10	0.90	0.87	0.91
Model 2	Three factors: N + EU, FRCC, OCB-CH	179.91	41	93.48**	0.14	0.79	0.72	0.80
Model 3	Two factors: N + FRCC + EU, OCB-CH	198.13	43	111.70**	0.15	0.77	0.71	0.77
Model 4	One factor: All variables combined	381.07	44	294.64**	0.22	0.50	0.37	0.51

N, narcissism; FRCC, felt responsibility for constructive change; EU, environmental uncertainty; OCB-CH, change-oriented OCB. **p < 0.01.

#### Common Method Variance Test

As in Study 1, we applied the Harman single-factor test to examine the level of CMV. The results showed that the variance of the first common factor accounted for was 30.26%, far below the 50% standard (Yong and Pearce, 2013). This indicates that there is no serious CMV problem among the measured variables.

#### Descriptive Analysis Results

Table 5 presents the means, standard deviations, zero-order correlations, and internal consistency alphas for all the variables. Consistent with our hypotheses, narcissism was positively and significantly related to FRCC (*r* = 0.41, *p* < 0.01), and FRCC was positively and significantly related to OCB-CH (*r* = 0.19, *p* < 0.05).

**TABLE 5:** Study 2 descriptive statistics and correlation matrix.

	*M*	*SD*	1	2	3	4	5	6	7	8	9
(1) Gender (0 = male, 1 = female)	0.39	0.49									
(2) Age	31.77	6.52	–0.09								
(3) Education level	1.91	0.75	0.06	0.26**							
(4) Organization tenure	10.39	6.61	–0.05	0.84**	0.12						
(5) Company type (0 = Company A, 1 = Company B)	0.41	0.49	–0.01	–0.12	–0.02	–0.12					
(6) Narcissism	4.22	0.64	–0.01	0.05	–0.01	0.05	−0.16*	(0.92)			
(7) Environmental uncertainty	4.57	1.39	0.09	–0.01	0.08	–0.03	–0.12	0.21**	(0.72)		
(8) Felt responsibility for constructive change	4.26	0.89	–0.002	0.003	–0.01	–0.03	–0.13	0.41**	0.08	(0.63)	
(9) Change-oriented OCB	3.30	0.86	0.12	0.22**	0.05	0.21**	−0.14^†^	0.19*	0.06	0.19*	(0.86)

^†^p <0.1; *p < 0.05; **p < 0.01. Coefficient alphas are reported in parentheses along the diagonal.

*Employee education level: 1, junior college or lower; 2, bachelor’s degree; 3, master’s degree; 4, PHD.*

#### Hypothesis Testing

**FIGURE 3:**
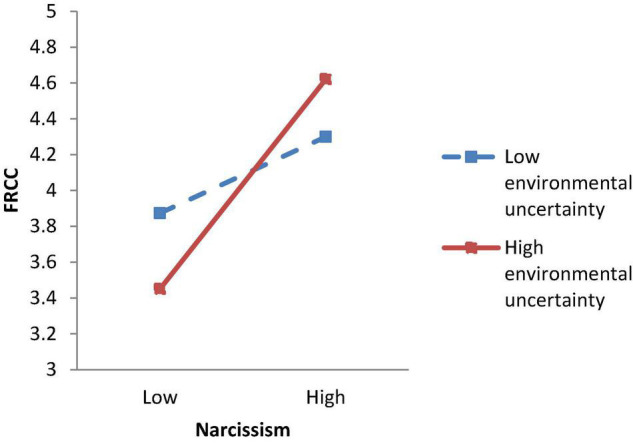
Study 2 moderating effect of environmental uncertainty on the relationship between narcissism and felt responsibility for constructive change.

We applied regression analysis to test the hypotheses. Supporting Hypothesis 1, Model 8 of Table 6 showed that the interaction term between narcissism and environmental uncertainty was significantly related to FRCC (γ = 0.21, *p* < 0.01). Figure 3 and simple slope tests showed that the relationship between narcissism and FRCC was stronger when environmental uncertainty was high (simple slope = 0.92, *p* < 0.01) and weaker when environmental uncertainty was low (simple slope = 0.35, *p* < 0.01). Hypothesis 2 proposed a positive relationship between employee FRCC and OCB-CH. As demonstrated in Model 10 of Table 6, FRCC was significantly related to OCB-CH (γ = 0.18, *p* < 0.05).

**TABLE 6:** Study 2 regression results.

	Felt responsibility for constructive change						Change-oriented OCB			
	Model 6		Model 7		Model 8		Model 9		Model 10	
	b	SE	b	SE	b	SE	b	SE	b	SE
***Control variables***										
Gender	0.001	0.14	0.01	0.13	0.05	0.13	0.25^†^	0.14	0.25^†^	0.13
Age	0.01	0.02	0.01	0.02	0.01	0.02	0.03	0.02	0.03	0.02
Education	–0.02	0.10	–0.02	0.09	–0.04	0.09	–0.02	0.09	–0.02	0.09
Organization tenure	–0.02	0.02	–0.02	0.02	–0.02	0.02	0.004	0.02	0.01	0.02
Company type	–0.23	0.14	–0.12	0.13	–0.13	0.13	–0.18	0.13	–0.14	0.13
***Independent variables***										
Narcissism			0.56**	0.10	0.63**	0.10				
Environmental uncertainty					–0.02	0.05				
***Mediator***										
Felt responsibility for constructive change									0.18*	0.07
***Interaction***										
Narcissism × environmental uncertainty					0.21**	0.07				
*R* ^2^	0.02		0.17		0.22		0.08		0.12	
Adj. *R*^2^	–0.01		0.14		0.18		0.05		0.08	
Δ*R*^2^			0.15**		0.04*				0.03*	
*F*	0.65		5.57**		5.48**		2.84*		3.45**	

^†^p <0.1; *p < 0.05; **p < 0.01.

Hypothesis 3 proposed an interaction effect between narcissism and environmental uncertainty on OCB-CH via FRCC. As in Study 1, estimation of the conditional indirect effects revealed that the indirect effect of narcissism on OCB-CH via FRCC was stronger when environmental uncertainty was high (*effect* = 0.14, 95%CI [0.02, 0.34]) and weaker when environmental uncertainty was low (*effect* = 0.05, 95%CI [0.001, 0.15]), and the difference was significant (*effect* = 0.09, 95%CI [0.01, 0.24]) Thus, Hypothesis 3 was supported.

#### Study 2 Discussion

Via a survey conducted in corporate settings during the COVID-19 pandemic, the results of Study 2 supported the hypothesis that employee narcissism and environmental uncertainty would have an interactive effect on OCB-CH via FRCC. Specifically, employee perceived environmental uncertainty strengthened the positive effect of narcissism on FRCC, and subsequently, the positive indirect effect of narcissism on OCB-CH. It is thereby demonstrated that our findings are consistent across samples, and are generalizable.

## Discussion

The COVID-19 pandemic has brought unprecedented uncertainties to the workplace. Employees need to perform work beyond their formal job requirements, proactively cope with dynamic environments, and take the initiative to respond to uncertainties. It is thus critical to investigate how to promote employees’ OCB-CH. The results of the current study demonstrate that narcissism, long thought of as a “dark trait,” can indeed generate high levels of OCB-CH via employees’ FRCC, especially when environmental uncertainty is high.

### Theoretical Contributions

This research contributes to several streams of literature. First, it extends the OCB-CH literature by uncovering a new antecedent. Previous literature on antecedents of OCB-CH has demonstrated that work context—including strong vision, innovative climate, supportive leadership (Choi, 2007), transformational leadership (Loìpez-Domiìnguez et al., 2013), and empowering leadership (Li et al., 2016)—can cultivate OCB-CH. However, it has been recommended that more attention be paid to the dispositional antecedents of OCB-CH (Seppälä et al., 2012), as internal characteristics can strongly motivate behavior (Hui et al., 2000). Personal characteristics like self-efficacy (Loìpez-Domiìnguez et al., 2013), sense of power (Seppälä et al., 2012), promotion focus (Simo et al., 2016), and psychological empowerment (Choi, 2007) have been found to be associated with OCB-CH. Based on these findings, we proposed and found that narcissism—characterized by an inflated self-view, a need for power and self-affirmation, and greater confidence regarding uncertainty and less fear regarding risk—was positively associated with OCB-CH. These findings enrich our understanding of the antecedents of OCB-CH.

Notably, OCB-CH is, by definition, different from other OCBs in terms of its emphasis on breaking the *status quo*, challenging routines, and disrupting social relationships to stimulate change (Choi, 2007). To date, however, there is still a lack of evidence to support the difference between OCB-CH and other OCBs. Our research provides some indirect evidence by demonstrating that narcissism is positively related to OCB-CH, in contrast to Webster and Smith’s (2019) finding that narcissism is negatively related to OCB.

Second, our research contributes to the narcissism literature by revealing its positive outcomes. Management researchers, following personality psychologists, have suggested that narcissism is a common personality trait (Campbell et al., 2005). In fact, it has been found that narcissism is particularly prevalent in younger adults today, who have been described as “Generation Me” (Twenge, 2013; Braun, 2017). Narcissism was originally considered as a “dark” personality trait, as it implies self-interest, arrogance, and entitlement, and is associated with negative, aggressive, and counterproductive behaviors that impede organizational functioning (Braun, 2017). However, in practice, many leaders are characterized as narcissistic, and have self-enhancing tendencies, engage in impression management, and strive for recognition and success. They are also considered to have high self-confidence and charisma. All of these elements are related to narcissism but can also contribute to leader emergence and leadership effectiveness (Brunell et al., 2008; Campbell and Campbell, 2009). Therefore, as narcissists thrive in the leadership domain, scholars have begun to notice the bright side of narcissism. For example, narcissism has been found to be positively related to mental toughness and performance under stress (Papageorgiou et al., 2019), information search effort and creativity (Zhou et al., 2019), occupational self-efficacy, career engagement, career success (Hirschi and Jaensch, 2015), and enhanced performance after ego threat (Nevicka et al., 2016). By exploring how and when narcissistic employees engage in OCB-CH from a TAT perspective, we extend this line of research in two ways. First, our research enriches the range of the possible positive consequences of narcissism. In particular, we found that narcissism may not only benefit individuals themselves through enhancing performance, creativity (Nevicka et al., 2016), and career success (Hirschi and Jaensch, 2015) but may also benefit organizations by promoting citizenship behaviors. Second, our research identifies important contextual factors that may trigger the positive aspects of narcissism. In particular, we found that in addition to performing well under stressful conditions (Wallace and Baumeister, 2002; Nevicka et al., 2016), narcissists may also thrive in uncertain environments. We considered a more specific aspect of environmental uncertainty (i.e., technology uncertainty) in Study 2 and replicated the findings in Study 1. In doing so, we respond to the call to further examine contingencies in strengthening the relationship between “dark traits” and “bright outcomes” (Spain et al., 2014, p. 14).

This finding also has implications concerning the long-discussed issue of narcissism and adaptability. Echoing most previous findings that, within the range of normal personality variation, narcissistic grandiosity is positively associated with adaptive psychological functioning and mental health (Jauk and Kaufman, 2018), we found that, in uncertain and volatile environments, grandiose narcissists demonstrate high adaptivity and react more positively and proactively than other individuals. The current study used samples of average employees to study the effects of narcissism, and the results concurred with the proposition in previous work that, for a subsample with low to moderate levels of grandiosity, grandiose narcissism’s positive association with self-esteem and dominance preference and negative association with fear of rejection and failure generate the “happy face” of this dark personality trait (Rose, 2002, p. 388).

Additionally, our research found a new mechanism (i.e., FRCC) that could explain the effects of narcissism on OCB-CH. Although studies have begun to investigate the positive influence of narcissism on desirable outcomes, few have revealed its underlying motivational mechanism (Mao et al., 2020). We thus advance the understanding of how and when narcissism can motivate employees to engage in OCB-CH.

Finally, our research contributes to the FRCC literature by finding a new personality antecedent. Previous studies have largely focused on work context, suggesting work design (Fuller et al., 2006), innovative climate (Loìpez-Domiìnguez et al., 2013), servant leadership (Arain et al., 2019), and responsible leadership (Han et al., 2019) as antecedents of FRCC, while neglecting the role of personality. This is an important omission, as individual differences should generate different motivations for creating change (Fuller et al., 2012). In addition, this study took an interactionist perspective and found that narcissism and environmental uncertainty interactively impact FRCC, responding to the proposition that FRCC is a function of both context characteristics and individual differences (Fuller et al., 2006).

### Practical Implications

The COVID-19 pandemic has posed great challenges for employees in the workplace. Today’s employees need to proactively respond to these changes, and our research provides some suggestions. First, managers should take a comprehensive and interactionist approach when considering narcissism. This study found that narcissism’s positive effects on FRCC and OCB-CH were stronger when organizations faced high levels of environmental uncertainty. This suggests that managers and organizations should have comprehensive understandings of the different personality traits of employees and also consider the external environment. OCB-CH is critical during periods of rapid environmental change, but most employees—and even leaders—tend to be more willing to maintain the *status quo* and engage less in OCB-CH under such circumstances (Dan et al., 2017). The average compliant and agreeable employee is not sufficient for coping with uncertainty and instigating change. Narcissists perform better during crises (Wallace and Baumeister, 2002) and are likely to be the first movers with regard to change and reform. Therefore, when organizations are facing changes and uncertainties, hiring narcissistic employees may be a viable strategy, and managers should also be attentive to preserving these employees’ self-efficacy by encouraging them to voice their opinions and take initiative.

Another practical implication of this study concerns the importance of employees’ felt responsibility for change. In both studies, FRCC was found to be positively related to OCB-CH, meaning that employees will act proactively to improve work practices or even break old rules and innovate if their sense of responsibility can be mobilized. Therefore, organizations should foster a sense of responsibility among their employees so that employees will feel motivated and obligated to engage in more positive behaviors. For instance, organizations can invite employees to participate in decision-making, offer them more autonomy and influence, cultivate their sense of ownership over their work to promote their sense of responsibility, and encourage them to identify and implement changes and improvements.

### Limitations and Future Directions

Our research has several limitations that merit future exploration. First, the cross-sectional nature of our data collection procedures could raise concerns regarding CMV. To mitigate these concerns, we used procedural and statistical remedies (Podsakoff et al., 2003). First, in Study 2, we used random identification numbers so that supervisors’ and subordinates’ responses could be matched to protect participants’ confidentiality. With this design, we aimed to reduce participants’ apprehension regarding the evaluation and encourage them to answer questions as objectively as possible. Second, we used multi-source method to measure key variables from supervisors and subordinates, with supervisors providing assessments of their subordinates’ OCB-CH. The use of other-rater (supervisor) reports, rather than employees’ self-ratings, to measure behavioral results provides a more reliable indication of narcissistic employees’ actual contributions to work (Mao et al., 2020). Finally, we performed Harman’s single-factor test and CFA to test the data for the absence of significant CMV at the level of the statistical results. Thus, while there is reason to believe that CMV does not confound our interpretations, the possibility must nonetheless be acknowledged.

Another limitation of this study is that cross-sectional studies may not provide clear information about causal relationships. Previous research has consistently shown that personality traits are strong predictors of contextual performance (Motowidlo and Van Scotter, 1994; Van Scotter and Motowidlo, 1996; LePine and Van Dyne, 2001). Based on TAT and logical reasoning, the current research proposed and revealed that narcissism was positively related to OCB-CH via FRCC, especially in uncertain environments. However, we are still unable to draw conclusions regarding causal relationships. In addition, both of the studies in this research were conducted under the specific conditions of the COVID-19 outbreak and its duration in China. Therefore, time is limited with regard to investigating the influence of the environmental uncertainty prompted by the COVID-19 pandemic, making a longitudinal study more difficult. We nevertheless encourage future research to complement the current study by using experiments or longitudinal field studies to better address causal inferences.

Another point worth noting is that, although the current research found a positive influence of narcissism on OCB-CH, these results are limited to several boundary conditions. First, our results are based on grandiose narcissism and may not be generalizable to other types of narcissism. For example, vulnerable narcissism, which is the pathological aspect of narcissism (Back et al., 2013), represents a defensive and insecure form of narcissism. When facing an uncertain environment, people with vulnerable narcissistic traits will likely not use self-enhancing strategies to promote change. Rather, they may demonstrate a reactive and resistant posture to obscure feelings of incompetence, anger, and anxiety (Miller et al., 2011) and are therefore unlikely to engage in OCB-CH. In a similar vein, in narcissistic rivalry, people maintain a grandiose self-image based on a defensive and avoidant motivation. They strive to prove their superiority over others and are afraid of losing status and admiration as a result of any changes (Krizan and Herlache, 2018). Therefore, people engaged in narcissistic rivalry are less likely to exhibit OCB-CH. Gebauer and colleagues have even recently divided grandiose narcissism into two sub-types: agentic narcissism and communal narcissism (Gebauer and Sedikides, 2018; Nehrlich et al., 2018; Rentzsch and Gebauer, 2018). Our research focuses on agentic narcissism, which is the traditional form of grandiose narcissism and is measured using the NPI-16. Agentic narcissists care about their agentic attributes and seek attention regarding their power, status, intelligence, and creativity (Campbell and Campbell, 2009). OCB-CH can satisfy these needs and thus is likely to be associated with agentic narcissism. However, communal narcissists pay attention to interpersonal relationships and are inclined to overstate their warmth, closeness, helpfulness, and love (Campbell and Campbell, 2009; Gebauer et al., 2012). While these characteristics may generally enable people to demonstrate citizenship behavior toward leaders or coworkers, they seem to be irrelevant to OCB-CH, given the change-oriented and agentic nature of this type of citizenship behavior. In fact, a previous study found that, although communal narcissists believed in their own extraordinary prosociality, there was no significant relationship between communal narcissism and objective prosociality (Nehrlich et al., 2018). In sum, it would be intriguing to explore the possible bright sides of other types of narcissism using alternative measures to the NPI-16.

Further, individuals in our sample showed moderate levels of narcissism (Study 1: *M* = 4.34, *SD* = 0.82; Study 2: *M* = 4.22, *SD* = 0.64). These results are consistent with Chinese culture, in which people are expected to behave modestly, as well as with the findings of previous studies using Chinese samples (e.g., Zhang et al., 2017). It would be interesting to test our model in other cultural contexts where there is a larger proportion of individuals with high levels of narcissism. Such contexts would provide the opportunity to explore whether grandiose narcissism and OCB-CH have an inverted U-shape relationship. Such a finding would mean that the positive relationship found in this research is limited to low to moderate levels of narcissism and that, when narcissism is sufficiently high, even grandiose narcissism can negatively impact OCB-CH (Jauk and Kaufman, 2018).

The results in our research are also limited to short-term effects. The questions of whether the relationship between narcissism and OCB-CH lasts in the long run and whether it indeed leads to better performance and organizational function warrant further consideration. Organizational change is an ongoing process, but existing research suggests that narcissists prioritize immediate need satisfaction and personal benefit over long-term relationships (Campbell and Campbell, 2009). The current research supports the short-term benefits of narcissism in initiating change and improvement but cannot be used to infer long-term benefits. Indeed, previous studies suggest that narcissists can induce long-term costs due to characteristics such as decreased engagement (Robins and Beer, 2001), overconfident decision-making, aggression, and volatile performance (Campbell and Campbell, 2009). Therefore, future research should study the long-term results of narcissists’ behaviors and compare the short-term benefits of narcissism with its long-term costs. For instance, multi-wave longitudinal studies are needed to investigate narcissists’ psychological states, behaviors, and influence on organizations.

Another related question not answered by this study is how narcissists will react if the change they would like to initiate is not implemented or fails to work, both of which are very common scenarios in organizations. We speculate that the situation would be different for grandiose and vulnerable narcissists. We expect that grandiose narcissists, who are the focus of the current study, will react positively or aggressively following negative feedback. Previous research suggests that, within the range of normal personality variation, grandiose narcissism is indicative of adaptive psychological functioning (Jauk and Kaufman, 2018) and is positively related to openness and negatively related to neuroticism (Weiss and Miller, 2018). Therefore, in the face of setbacks, grandiose narcissists are less likely to feel depressed or pessimistic. In fact, Nevicka et al. (2016) found that non-clinical grandiose narcissists tended to react aggressively after they received information that did not match their high self-views, displayed greater willingness to perform challenging tasks and performed better on creative tasks. For vulnerable narcissists, in contrast, setbacks may provoke more negative reactions. Vulnerable narcissism has been found to be positively related to neuroticism, greater psychological distress and negative emotions (e.g., anxiety and shame), low self-esteem and feelings of inferiority, and hostile interpersonal behaviors (Weiss and Miller, 2018). Vulnerable narcissists are thus attentive to others’ feedback regarding their behaviors; non-ideal results would enhance their feelings of inadequacy and incompetence as well as their negative affect (Miller et al., 2011), which may have negative consequences in the workplace. Future research can further investigate this question.

## Conclusion

Our study investigates whether, how, and when narcissism is related to OCB-CH, a type of unconventional and challenging citizenship behavior that is especially preferred in today’s business environment. We identify a “bright side” of narcissism and find that individual narcissism interacts with the environmental uncertainty prompted by the COVID-19 pandemic to have a positive influence on OCB-CH via FRCC. The association between narcissism and OCB-CH via FRCC is stronger when environmental uncertainty is higher. These results offer a more comprehensive understanding of this “dark” trait by revealing the critical boundary condition and underlying mechanism of its positive effect. This research also extends the literature on OCB-CH and FRCC by revealing a new antecedent (i.e., narcissism).

## Data Availability Statement

The raw data supporting the conclusions of this article will be made available by the authors, without undue reservation.

## Ethics Statement

Ethical review and approval was not required for the study on human participants in accordance with the local legislation and institutional requirements. Written informed consent for participation was not required for this study in accordance with the national legislation and the institutional requirements.

## Author Contributions

YL: conceptualization, data collection, data analysis, funding acquisition, project administration, writing – original draft, and writing – review and editing. HZ: conceptualization, data collection, funding acquisition, project administration, writing – original draft, and writing – review and editing. JL: conceptualization, writing – original draft, and writing – review and editing. XZ: conceptualization and data collection. All authors: contributed to the article and approved the submitted version.

## Conflict of Interest

The authors declare that the research was conducted in the absence of any commercial or financial relationships that could be construed as a potential conflict of interest.

## Publisher’s Note

All claims expressed in this article are solely those of the authors and do not necessarily represent those of their affiliated organizations, or those of the publisher, the editors and the reviewers. Any product that may be evaluated in this article, or claim that may be made by its manufacturer, is not guaranteed or endorsed by the publisher.

## Footnotes

1
http://www.quantpsy.org/medmc/medmc111.htm
